# Cathelicidin in Urinary Tract Diseases: Diagnostic, Prognostic and Therapeutic Potential of an Evolutionary Conserved Antimicrobial Protein

**DOI:** 10.3390/medicina60122015

**Published:** 2024-12-06

**Authors:** Iva Sorić Hosman, Andrea Cvitković Roić, Ivana Vuković Brinar, Tonko Gulin, Marijana Ćorić, Dunja Rogić, Ana Lončar Vrančić, Lovro Lamot

**Affiliations:** 1 Department of Pediatrics, Zadar General Hospital, 23000 Zadar, Croatia; 2 Department of Nephrology and Urology, Clinic for Pediatric Medicine Helena, 10000 Zagreb, Croatia; 3 School of Medicine, Josip Juraj Strossmayer University of Osijek, 31000 Osijek, Croatia; 4 School of Medicine, University of Rijeka, 51000 Rijeka, Croatia; 5 Department of Nephrology, Hypertension, Dialysis and Transplantation, University Hospital Centre Zagreb, 10000 Zagreb, Croatia; 6 School of Medicine, University of Zagreb, 10000 Zagreb, Croatia; 7 Department of Nephrology and Dialysis, Sestre Milosrdnice University Hospital Centre, 10000 Zagreb, Croatia; 8 Department of Pathology and Cytology, University Hospital Centre Zagreb, 10000 Zagreb, Croatia; 9 Faculty of Pharmacy and Biochemistry, University of Zagreb, 10000 Zagreb, Croatia; 10 Department of Laboratory Diagnostics, University Hospital Centre Zagreb, 10000 Zagreb, Croatia; 11 Division of Nephrology, Dialysis and Transplantation, Department of Pediatrics, University Hospital Centre Zagreb, 10000 Zagreb, Croatia

**Keywords:** cathelicidin, LL 37, urinary tract infection, urinary tract disease, biomarker

## Abstract

Despite being one of the most common infectious diseases, urinary tract infections (UTIs) still represent a challenge for clinicians to diagnose and treat, especially in the era of growing antibiotic resistance among uropathogenic bacteria. Recent studies investigating the pathophysiology of UTIs have discovered the prominent role of antimicrobial peptides in the urinary tract defense system. Cathelicidin is an evolutionary conserved antimicrobial peptide encoded by one single gene in humans. Except for being stored in neutrophil cytoplasmic granules, cathelicidin is produced by uroepithelial cells rapidly upon contact with a uropathogen, even before leukocytes invade the urinary tract. In addition to its bactericidal effect, cathelicidin acts as a chemoattractant for multiple immune cells and a potent inductor of numerous cytokine synthesis. Such a crucial role in the initial pathogenesis of a UTI makes cathelicidin a potential biomarker for an early UTI diagnosis. Indeed, multiple studies over the last two decades have proved the potential clinical utility of cathelicidin as a UTI diagnostic biomarker. Furthermore, since patients after the resolution of a UTI have been found to express a lower urinary cathelicidin level than healthy controls, decreased cathelicidin levels have been suggested as a risk factor for developing UTI recurrence. Therefore, measuring cathelicidin levels in urine might help in distinguishing patients with a higher risk for a recurrent UTI. Interestingly, except in UTIs, cathelicidin has also been evaluated in other urinary tract diseases and proposed as a biomarker for diagnosing severe vesicoureteral reflux (VUR) and for recognizing renal scar development in patients with VUR. Finally, a prominent role in UTI pathogenesis also makes cathelicidin an attractive therapeutic target for treating UTIs and, lately, different therapeutic agents up-regulating cathelicidin expression have been investigated in this matter. Therefore, the present review aims to summarize the current body of knowledge on the diagnostic, prognostic and therapeutic potential of cathelicidin in urinary tract diseases. For this purpose, three databases (Scopus, Medline and Web of Science) were extensively searched to cover all the published articles. This exhaustive review will update clinicians on the contemporary state of knowledge about the potential clinical utility of cathelicidin in urinary tract diseases and hopefully encourage further research, resulting in improvement in the current management of urinary tract diseases.

## 1. Introduction

Urinary tract infections (UTIs) are one of the most common infectious diseases in humans, affecting more than 150 million people annually [1]. Despite its high incidence, diagnosis of a UTI is still based on the result of urine culture and therefore is delayed for days since the first symptoms appear. Furthermore, in addition to the difficulties in diagnosing a UTI, predicting its recurrence is an ongoing challenge for clinicians. Finally, although antibiotics are the primary treatment for UTIs, the rising issue of bacterial resistance to multiple drugs and their questionable effectiveness in preventing recurrent UTIs highlight the need for new therapeutic strategies to treat and prevent UTIs in both children and adults [2,3,4].

The pathogenesis of UTI has been extensively explored during the last few decades. Emerging evidence suggests that UTI is a product of a complicated host–bacteria interaction and that innate immunity has a crucial role in maintaining homeostasis and protecting the urinary tract from invading uropathogens [5]. Innate defense in the urinary tract greatly depends on local production of antimicrobial peptides (AMPs) [6]. One of the most prominent antimicrobial peptides defending the urinary tract from bacterial invasion is uroepithelium-derived cathelicidin [7,8]. Therefore, a growing number of studies have been exploring its potential for clinical use in previously described problems of the diagnosis, prognosis and prevention of UTIs.

Cathelicidin is constitutively expressed in a small amount in renal epithelial cells, but a larger portion of cathelicidin is newly synthesized and released into the urine upon contact with uropathogenic bacteria [8]. This rapid increase in cathelicidin expression by bacterial component (pathogen-associated molecular patterns, PAMPs) activation of pattern recognition receptors (PRRs) on uroepithelial cells seems to play a more important part in the urinary tract defence system than a later influx of neutrophils, which release cathelicidin from their cytoplasmic granules in an advanced stage of a UTI [8]. The cathelicidin antimicrobial peptide (*CAMP*) gene in uroepithelial cells is firstly translated to a propeptide named human cationic antimicrobial peptide 18 (hCAP-18), which is then cleaved by serine proteases to the active form called LL 37 (37 amino-acid-long chain beginning with two leucines). This active form of cathelicidin binds to the negatively charged bacterial membrane, inducing its rupture and bacterial cell lysis [9]. In addition to its bactericidal potential, cathelicidin has been found to have multiple immunomodulatory roles affecting both innate and adaptive immunity [10,11]. Namely, it can neutralize endotoxins such as lipopolysaccharides (LPSs), and consequently prevent LPS-induced uroepithelial apoptosis. Furthermore, cathelicidin also acts as a chemoattractant for neutrophils, monocytes and T cells and as a stimulator of cytokine production, mostly monocyte chemoattractant protein (MCP)-1, MCP-3, interleukin (IL)-8 and IL-6, through activation of the NF-κB-mediated induction of gene transcription [10,11,12,13].

**Figure 1:**
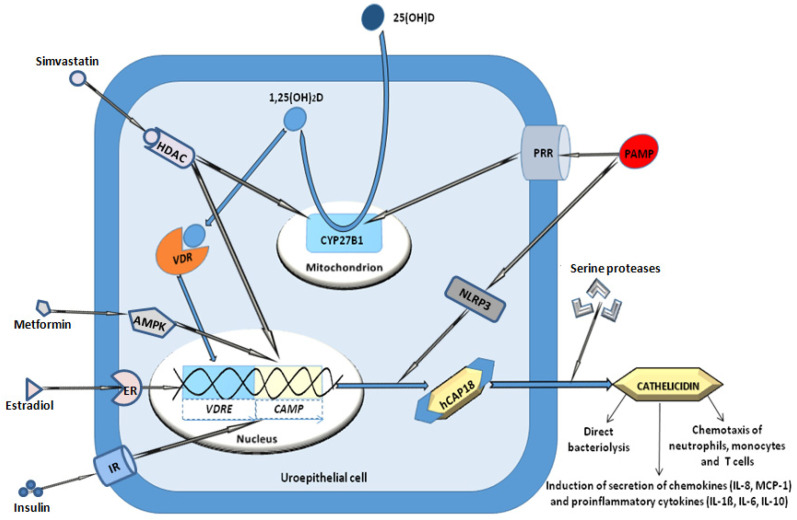
Processes of the induction, synthesis and secretion of cathelicidin in a uroepithelial cell. Pathogen-associated molecular patterns (PAMPs) on invading uropathogens activate pattern recognition receptors (PRRs). The subsequent downstream intracellular signalling induces mitochondrial 1α–hydroxylase (CYP27B1) for conversion of 25-hydroxy vitamin D (25(OH)D) to the active form 1,25-dihydroxy vitamin D (1,25(OH)_2_D). The active form binds to the vitamin D receptor (VDR) and the complex then translocates to the nucleus and binds to the vitamin D responsive element (*VDRE*) in the promotor region of the cathelicidin antimicrobial peptide (*CAMP*) gene, inducing its transcription. The precursor peptide, human cationic antimicrobial peptide of 18 kDa (hCAP18), is processed to cathelicidin by serine proteases. Insulin, estradiol, simvastatin, metformin and activation of NLPR3 also induce cathelicidin expression in uroepithelial cells. AMPK = 5’ adenosine monophosphate-activated protein kinase, ER = estrogen receptor, HDACs = histone deacetylases, IR = insulin receptor, NLRP3 = NOD-like receptor protein 3.

Aside from bacterial components, cathelicidin’s production in uroepithelial cells is stimulated by vitamin D. The inactive form 25-hydroxy vitamin D_3_ (25(OH)D_3_) is converted in renal and uroepithelial cells to the active metabolite 1,25-dihydroxy vitamin D_3_ (1,25(OH)_2_D_3_) by a mitochondrial 1α–hydroxylase called CYP27B1. The latter enzyme is up-regulated by intracellular downstream signalling of an activated PRR. Finally, 1,25(OH)_2_D_3_ binds to the vitamin D receptor (VDR) and then the complex translocates to the nucleus, where it induces *CAMP* gene expression by binding to the vitamin D responsive element (VDRE) in the *CAMP* gene promoter [14]. Consequently, cathelicidin synthesis is up-regulated, resulting in increased antibacterial activity [15]. Processes of the induction, synthesis and secretion of cathelicidin in uroepithelial cell are summarized in Figure 1.

In addition to the local cathelicidin production by urothelial cells during an acute UTI, vitamin D up-regulates cathelicidin expression in a wide range of cell types, including macrophages. VDR signalling is linked to the activation of cathelicidin and subsequent stimulation of autophagy and cytokine production as well as regulation of the crosstalk between innate and adaptive immunity. This vitamin D–cathelicidin axis seems to modulate the host response in various infective diseases and in different immune system disorders [16,17].

Given these recent discoveries, substantial efforts have been directed toward harnessing the clinical potential of cathelicidin to reduce the burden of UTIs, whether as a diagnostic, prognostic or therapeutic agent. Therefore, in the present review we aimed to summarize the most important findings from studies investigating the potential use of cathelicidin in urinary tract diseases.

## 2. Search Methods

**Figure 2:**
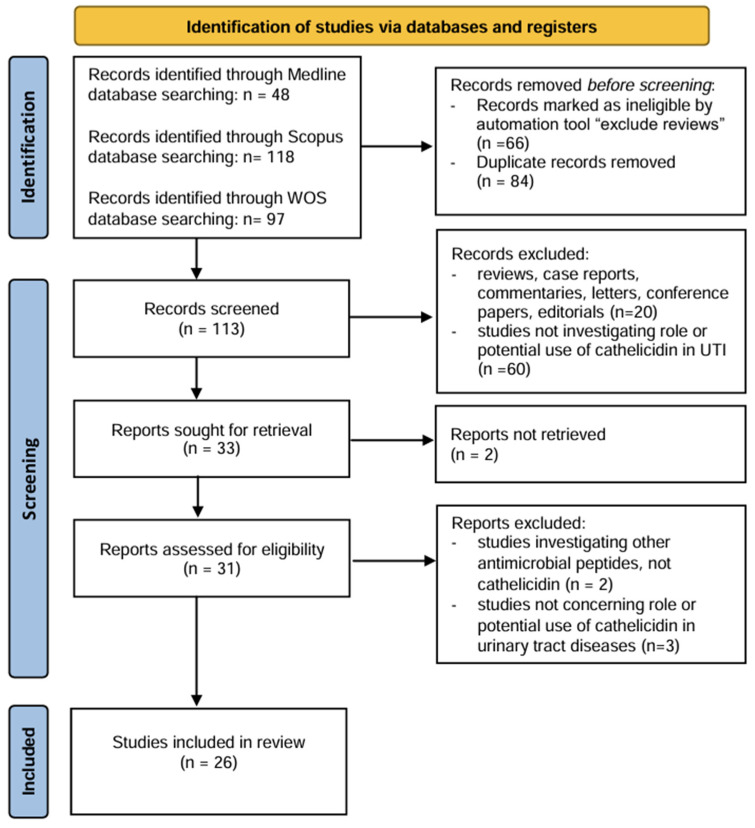
PRISMA flow diagram.

Although this is not a systematic review, in order to identify most of the original research papers concerning the role or potential use of cathelicidin in urinary tract diseases, an extensive literature search was conducted using the Pubmed, Scopus and Web of Science databases, in accordance with the published guidelines for writing reviews [18]. Articles in English published until 31 July 2024 with a combination of terms (“cathelicidin” OR “LL 37” AND “urinary tract”) in the title, abstract or keywords were sought in all of the three mentioned databases. Case reports, reviews, commentaries, conference papers and editorials were all excluded. Finally, only original research articles investigating cathelicidin’s diagnostic, prognostic or therapeutic role in urinary tract diseases were included in this review. The initial search of all three databases identified 263 articles, of which 197 remained after the use of an automated tool for review exclusion and 113 remained subsequent to duplicate removal. After title and abstract screening of the identified articles by two authors (LL and ISH), an additional 80 articles were excluded. Two of the remaining 33 articles were not fully available through our institutions. In the end, after evaluation the full-text articles for eligibility by two authors (LL and ISH) with the help of a third author (ACR) in the case of discrepancies, 26 full-text articles were incorporated and thoroughly analysed in the present review. The study selection process is presented step-by-step in a PRISMA flowchart (Figure 2).

## 3. Results

### 3.1. Cathelicidin as a Diagnostic Biomarker for Urinary Tract Diseases

Based on the elucidated pathways of the local uroepithelial production of cathelicidin during an acute UTI, the first clinical studies regarding cathelicidin’s role in the urinary tract were those investigating its potential as a diagnostic biomarker for UTIs.

Chromek et al. [7] were the first to provide evidence of abrupt cathelicidin synthesis during an acute UTI. Firstly, they demonstrated that children with acute cystitis or pyelonephritis (n = 29) have notably elevated urinary cathelicidin levels as opposed to healthy controls (n = 28). Secondly, as a small positive correlation between urinary cathelicidin concentration, urinary leukocyte count and urinary neutrophil enzyme myeloperoxidase (MPO) level was found, the investigators suggested an additional origin of urinary cathelicidin, other than neutrophil cytoplasmic granules. Indeed, they proved that uroepithelial cells are positive for both cathelicidin mRNA and the cathelicidin peptide during an acute UTI. A substantial increase in the synthesis and secretion of cathelicidin during bacterial infection was corroborated in vitro in human renal and uroepithelial cells incubated with uropathogenic *Escherichia (E.) coli* (UPEC) for 24 h. Finally, the role of cathelicidin in protecting against urinary tract infection was assessed in mice with an intact and those with a deleted cathelicidin-related antimicrobial peptide (*CRAMP*) gene. The end point was the number of bacteria attached to the bladder 1 h after the infection, a time point before neutrophils invade the urinary space. The number of adhering bacteria was greater in the *CRAMP* knockout mice. Taken all together, Chromek et al. [7] proved that cathelicidin could be utilized as a diagnostic biomarker for an acute UTI based on its local synthesis in the uroepithelium during an acute infection, which takes place even before leukocyte invasion into the urinary tract.

In contrast, Danka and Hunstad [19] found that *CRAMP*-deficient mice demonstrated lower bladder bacterial loads at multiple time points and recovered more quickly from cystitis than those with the intact gene. *CRAMP*-deficient mice exhibited an attenuated immune response to infection and consequently less tissue damage and accelerated epithelial restoration. Their findings support cathelicidin’s role in the activation of the host immune response to uropathogens, which might be a protective response but might also aggravate local inflammation with consequent uroepithelial destruction. Oottamasathien et al. [20] supported the proinflammatory role of cathelicidin in their research on mouse models. In their experiment, cathelicidin induced dose-dependent inflammation through mast cell recruitment. Given these contrary findings, further studies of cathelicidin’s role in UTI pathogenesis are needed.

Regardless of cathelicidin’s exact role in an acute UTI, clinical studies mostly confirmed its up-regulation during an acute UTI. Nielsen et al. [21] reported that urinary cathelicidin levels are significantly higher during an acute UTI than post-infection in otherwise healthy premenopausal women. Interestingly, the post-infection cathelicidin levels were significantly lower in patients who had a UTI than those of healthy controls who had never had a UTI, suggesting that low cathelicidin production might be associated with UTI development. Thus, the authors indicated that urinary cathelicidin concentration might be both a diagnostic and predictive biomarker for UTIs. However, whether low post-infection values of urinary cathelicidin concentration are a predisposing factor or a time-limited consequence of a previous UTI should be the question of interrogation in future prospective studies. Van der Starre et al. [22] confirmed significantly elevated urinary cathelicidin levels in children with UTI compared to healthy controls, though the cathelicidin concentration did not correlate with bacteremia, making it inapplicable as a bacteremia biomarker. The increased urinary cathelicidin levels in the UTI group were noted despite a high vitamin D insufficiency rate, probably because the same rate of vitamin D insufficiency was found in the control group (86 and 87%, respectively).

On the contrary, Ovunç Hacıhamdioglu et al. [23] showed no significant differences in the levels of urinary LL-37 between children with UTI (n = 36) and the control group (n = 38). Furthermore, the study by Ovunç Hacıhamdioglu et al. proved that children with insufficient vitamin D plasma levels are not able to elevate their urinary cathelicidin level during an acute UTI, which corroborated assumptions from previous studies [14,15] that vitamin D level is essential for urothelial cathelicidin production. This observation might explain the lack of difference in cathelicidin levels between the UTI and the control group in this study, since a larger proportion of vitamin D insufficiency in the UTI group (87.8%) was found compared to healthy controls (41.7%). This might also be the reason for finding cathelicidin inadequate for predicting a positive urine culture in the case of suspected UTI in a previous study by Caterino et al. [24]. The latter study included 40 patients with suspected UTI, out of whom only 13 eventually had a positive urine culture. Initial cathelicidin levels were not correlated with urine culture positivity. Since cathelicidin expression is vitamin D-dependent, these results might have been impacted by a high proportion of patients with vitamin D insufficiency (72%). Furthermore, most of the patients (58%) were older than 65 years and most of them had different comorbidities, including diabetes, cardiovascular disease and cancer, which might have also impacted results.

More recently, several studies [25,26,27,28,29] have confirmed cathelicidin as a reliable diagnostic biomarker for UTI. Babikir et al. [25] demonstrated that cathelicidin could act as an early diagnostic biomarker for UTI (before urine culture results). In their case–control study involving children and adults, both serum and urinary cathelicidin levels were notably increased in the UTI group (n = 87) compared to healthy controls (n = 87). Awadallah et al. [26] validated that the urinary cathelicidin level differs between patients with culture-positive UTI (proven UTI group, n = 50) and those with UTI symptoms but with negative urine cultures (suspected UTI group, n = 20) as well as from healthy controls (n = 20). Furthermore, the diagnostic performance of cathelicidin was assessed in a ROC curve analysis, which confirmed its ability to differentiate proven UTI from suspected UTI with an AUC of 0.982. Alhamedy et al. [27] and Ali et al. [28] corroborated the utility of both urinary and serum cathelicidin levels for UTI diagnosis in their studies of adult UTI patients. Krivošikova et al. [29] demonstrated that children under the age of two with a first acute febrile UTI caused by *E. coli* have higher urinary cathelicidin levels compared to healthy controls. Moreover, cathelicidin positively correlated with leukocyturia. Limitations of the study include cross-sectional design as well as a lack of age and sex matching between cases and controls (the UTI group contained two-thirds female children, while healthy controls consisted of approximately half girls and half boys).

Georgieva et al. [30] investigated vitamin D and cathelicidin levels in the plasma of children one month after an acute UTI (n = 76) compared with those with congenital hydronephrosis but without a history of UTI (n = 44). They found lower levels of both plasma vitamin D and plasma cathelicidin in the UTI group. This is in concordance with previously reported low post-infectious urinary cathelicidin levels in adults [21]. Furthermore, 76 children from the UTI group were prospectively followed for one year, but they found no correlation between initially measured plasma cathelicidin level and UTI recurrence. However, the study sample in this longitudinal part of the study was small (only 21 had recurrence within one year). Furthermore, cathelicidin plasma level was measured one month after the first UTI and urinary cathelicidin concentration was not measured at all, so further prospective cohort studies are needed to investigate cathelicidin as a UTI recurrence predictor.

Lezhenko and Zakharchenko [31] aimed to identify factors mostly associated with the risk of developing chronic (recurrent) UTIs in children. Their study encompassed 77 children with a primary UTI, of whom 34 developed chronic UTI. They did not find serum cathelicidin level to be correlated with chronic UTI development. However, serum vitamin D concentration was found to be strongly associated with development of chronic inflammatory diseases of the urinary system in children. Since local production and urinary cathelicidin level during UTI depend on serum vitamin D level, further studies are needed to investigate the urinary cathelicidin level in an acute UTI as a predictor of developing recurrent UTIs. The authors did not specify how the diagnosis of chronic UTIs was made (how long the follow-up after the first UTI lasted and what the criteria were for diagnosis of chronic UTIs). Furthermore, the epidemiological factors of the included subjects, namely sex and age, and the difference between the studied groups regarding these factors was not taken into account in the statistical analysis. Therefore, further studies on this important matter are warranted.

Except in an acute UTI, cathelicidin has been proved to be elevated in obstructive uropathy, even in unilateral cases (compared to the widely used creatinine level). Gupta et al. [32] found that urinary cathelicidin levels, as well as AMPs beta defensin 1 (BD-1), hepatocarcinomatous-intestine-pancreas/pancreatitis-associated protein (HIP/PAP) and neutrophil gelatinase-associated lipocalin (NGAL), are elevated in patients with unilateral ureteropelvic junction obstruction (UPJO) requiring surgery without an acute infection as compared to age- and sex-matched healthy controls. These results suggest that urinary AMP levels could be used as markers of urinary tract obstruction. The elevation of urothelial-derived AMPs in the setting of obstruction is probably a consequence of uroepithelial stress produced by obstructive uropathy. As a follow-up to this initial study, a subsequent study by the same investigator group [33] examined the expression of these AMPs after successful surgical correction of the obstruction in a subset of patients who were at least 6 months from surgical repair. However, in spite of the clinical improvement and signs of improved hydronephrosis on post-operative imaging, urinary cathelicidin levels did not alter significantly from those before the surgery. Therefore, cathelicidin did not prove to be a prognostic biomarker in the case of surgical correction of obstructive uropathy. The same group of AMPs was examined in a study of 36 children with neurogenic bladder and all of them were elevated, even in those without an acute infection, compared with healthy controls [34]. This is not surprising, since patients with neurogenic bladder have known urothelial abnormalities [35], and the evaluated AMPs are urothelially derived. However, within the group of children with neurogenic bladder, only NGAL differed between those with UTI and those with asymptomatic bacteriuria, proposing its diagnostic utility for UTI in children with neurogenic bladder. At the same time, urinary cathelicidin did not differ between the groups. Nevertheless, it must be noted that the number of patients in each group was small (UTI group n = 6; asymptomatic bacteriuria group n = 18) and groups significantly differed regarding gender and presence of VUR or hydronephrosis, which might have concealed cathelicidin’s ability to differ in the groups. So, additional studies are required to decide whether cathelicidin has a potential utility in this matter.

Colceriu et al. [36] proposed cathelicidin as a diagnostic marker of severe vesicoureteral reflux (VUR). In a cross-sectional study of 39 children with VUR and 39 healthy controls, urinary cathelicidin levels were higher in children with VUR, but the difference did not reach statistical significance. However, cathelicidin positively correlated with VUR severity (expressed as a cumulative scale from 1 to 10) and showed a good predictive value for diagnosing severe VUR (AUC 0.71). Furthermore, children with renal scarring (RS) had significantly lower urinary cathelicidin concentrations. Thus, cathelicidin could be a non-invasive biomarker of RS occurrence in patients with VUR. Patients with severe VUR probably have increased cathelicidin levels due to the innate immune reaction to mechanical uroepithelial destruction and proinflammatory state during RS development. On the other hand, decreased cathelicidin levels in the state of already developed RS might be a consequence of the urothelial destruction and therefore reduced local cathelicidin production. Since this is, to our knowledge, the only study of cathelicidin levels in children with VUR and RS, with a small number of patients (39 with VUR, among whom only 9 patients had RS), further studies are mandatory to corroborate these assumptions.

The studies analysed in this section are summarized in Table 1. Taken all together, cathelicidin definitely has potential as a diagnostic biomarker for an acute UTI, especially in patients with sufficient vitamin D levels, while a lack of cathelicidin increase in patients with vitamin D insufficiency must be taken with caution. In addition, patients with urinary tract obstruction or functional urinary tract abnormalities such as VUR might have elevated cathelicidin levels even without an acute infection, thus making cathelicidin’s utility as a diagnostic marker for UTI limited in such patients. On the other hand, studies evaluating urinary cathelicidin concentration as a prognostic UTI biomarker are lacking, since the only two studies investigating the predictive potential of cathelicidin for recurrent UTIs [30,31] measured only serum cathelicidin level. Those two studies did not find a correlation between serum cathelicidin level and UTI recurrence. Therefore, based on the previously explained pathogenesis of local cathelicidin production (Figure 1), we propose urinary cathelicidin level as a biomarker predictive of recurrent UTIs for future studies to investigate. Finally, only one study each correlated cathelicidin level with obstructive uropathy, neurogenic bladder and VUR. Therefore, urinary cathelicidin level as a measure of local defence system strength seems to be influenced by serum vitamin D level, with different states affecting the normal urine flow (obstruction, neurogenic bladder and VUR) and presence of an acute infection. Complex interactions between all these factors remain to be elucidated in future studies.

### 3.2. Cathelicidin as a Therapeutic Target in Urinary Tract Diseases

With the continuously rising number of multi-drug-resistant uropathogenic bacterial strains interfering with antibiotic therapy, alternative treatment strategies are mandatory. Despite the established protective role of cathelicidin in the urinary tract, the utility of its natural, linear form in a clinical setting has been precluded due to its toxicity to human cells [20,37] and proteolytic instability [38,39,40]. Therefore, stimulation of endogenous urothelial production of LL 37 by different agents and biologically stable peptides derived from LL 37 have been investigated and seem to be a promising option for UTI prevention and treatment. All studies analysed in this section are summarized in Table 2.

Herrting et al. [41] analysed bladder tissue from postmenopausal women for expression of cathelicidin, before and after a three-month period of supplementation with 25(OH)D_3._ Although vitamin D alone did not up-regulate cathelicidin in the serum or in bladder tissue of the women in this study, when the bladder biopsies were infected with UPEC, a significant increase in cathelicidin expression was observed after 25(OH)D_3_ supplementation. This observation was confirmed in human bladder cell lines. Vitamin D-treated bladder cells exerted an increased antibacterial effect against UPEC. Therefore, the authors proposed vitamin D supplementation for UTI prevention.

Except for vitamin D, estrogen supplementation has also been proved to induce cathelicidin production and has a potential utility in preventing UTI. Actually, Luthje et al. [42] reported increased cathelicidin production in urothelial cells from postmenopausal women before and after a 2-week period of estrogen supplementation. These results were also confirmed in a mouse UTI model, demonstrating enhanced antimicrobial capacity of the urothelium stimulated by estradiol. Altogether, these findings support the use of estrogen supplementation in postmenopausal women suffering from recurrent UTIs. More recently, Lindblad et al. [43] demonstrated that estrogen induction of uroepithelial cathelicidin synthesis is mediated through NLRP3-associated pathways. The authors investigated the involvement of NLRP3 in the UPEC-evoked release of cathelicidin from bladder epithelial cells after 6 h of stimulation. Human bladder epithelial cells and NLRP3-deficient cells were stimulated with the UPEC strain CFT073 and estradiol. The expression of most AMPs, including cathelicidin, was reduced in NLRP3-deficient cells, proposing NLRP3 as an essential factor for UPEC-induced up-regulation of cathelicidin expression. The ability of estradiol to induce an increased expression of AMPs was also abrogated in NLRP3-deficient cells. Based on these observations, authors suggested that NLRP3 regulates the expression of cathelicidin and other AMPs and affects estrogen signalling in bladder epithelial cells, leaving the question of NLRP3-based therapies for preventing or treating UTI for further investigation.

Induction of cathelicidin production by synthetic peptides has been investigated in several studies [44,45,46]. Wang et al. [44] demonstrated a protective role of cathelicidin-derived antimicrobial peptide Bmap-28 incorporated with polyurethane against bacterial biofilm formation of common pathogens for catheter-related UTI in vitro, thus suggesting its use in catheter-associated UTIs. Wnorowska et al. [45] proved that increased cathelicidin has a bactericidal effect against multi-drug-resistant *E. coli* strains when combined with ceragenins CSA-13 and CSA-131. White et al. [46] validated the therapeutic potential of synthetic peptide CD4-PP, designed by dimerization and backbone cyclization of the shortest antimicrobial region of human cathelicidin, in infected cultures of human uroepithelial cells. They observed that this peptide has a direct bactericidal effect by inducing membrane deformation and leakage in *E. coli* and *Pseudomonas (P.) aeruginosa*. Furthermore, CD4-PP treatment prevented the formation of a new biofilm and dissolved the mature biofilm created by *E. coli* and *P. aeruginosa*. On top of that, CD4-PP also induced production of cathelicidin by uroepithelial cells. The result was significantly reduced uropathogen survival when treatment was given at the start of infection. The investigators also demonstrated that urinary catheter pieces coated with saline fluid supplemented with CD4-PP reduced the attachment of *E. coli*, giving it a potential clinical application.

Mohanty et al. [47] explored the role of activation of hypoxia-inducible factor 1 (HIF-1) in infected uroepithelial cells in vitro as well as in mouse UTI models. They used hypoxia and 2-oxoglutarate analogue DMOG (HIF-hydroxylase inhibitor) for enhancing HIF-1, which is known to up-regulate the expression of cathelicidin by binding to its promoter sequence. After HIF-1 enhancement, the cell cultures and mice were infected with *E. coli* and showed a lower bacterial load than those not previously exposed to hypoxia and DMOG. Moreover, 7 days post-infection, the uroepithelium of all DMOG-treated mice was almost intact while in non-treated mice the investigators observed disruption of the uroepithelial lining with pronounced *E. coli* load.

Majhi et al. [48] demonstrated enhancement of innate immunity, including cathelicidin production, in uroepithelial cell cultures by metformin. Metformin pre-treated cells showed significantly improved killing of intracellular and extracellular bacteria, resulting in a lower bacterial load than non-treated cells. These results indicate that metformin prepares the uroepithelium to kill uropathogens more efficiently, and this is, at least partially, mediated by increased cathelicidin production. Furthermore, in a study by Luthje et al. [49] simvastatin was used for enhancing cathelicidin expression in bladder epithelial cells. In addition, simvastatin increased transcription of the vitamin D-activating enzyme CYP27B1, which, as previously explained, activates cathelicidin production. All of these findings are waiting for confirmation through in vivo studies.

Schwarz et al. [50] found that diabetic mice with a suppressed insulin receptor (IR) expression are more susceptible to UTIs and, furthermore, demonstrated that systemic IR activation reduces UTI susceptibility. Additionally, IR deletion in suprabasal cells increases UTI risk by suppressing the NF-κB stimulated increase in AMP expression. Altogether, these findings elucidate a critical role of the IR and downstream NF-κB activity in urothelial defenses against UPEC. On top of these findings, the authors observed suppressed expression of the IR and AMPs in exfoliated urothelial cells or urine samples from children with diabetes type 2 (n = 16) compared to healthy children (n = 15). Based on these observations, the authors concluded that urothelial insulin signalling has a role in UTI prevention through insulin receptor regulation of antimicrobial peptide expression. However, the generalizability of these conclusions is limited by the small, cross-sectional, single-centre nature of this study and requires corroboration in future studies.

Although most of the analysed studies rely on the supposed protective role of cathelicidin in the urinary tract, it must be noted that all of the agents used were tested against *E. coli*. Surprisingly, Patras et al. [51] demonstrated cathelicidin’s ineffectiveness in UTI caused by group B streptococcus (GBS) in streptozotocin-induced diabetic mice. In this UTI model, cathelicidin-deficient mice showed decreased bacterial burden and bladder mast cell recruitment compared to wild-type mice. Based on these observations, the authors suggested cathelicidin might be a contributor to increased susceptibility to GBS caused by UTI in diabetic patients.

Taken all together, although cathelicidin as a potent antimicrobial peptide represents an alternative UTI treatment to the traditional use of antibiotics due to its bactericidal activity, innate immunity enhancement and slower development of bacterial resistance, treatments targeting cathelicidin expression in the urinary tract demand validation in future prospective clinical studies.

## 4. Conclusions and Future Directions

Given the current challenges in the early diagnosis and prediction of UTI recurrence, new biomarkers are needed for both diagnosing UTIs and anticipating their recurrence. Based on the published literature, urinary cathelicidin concentration correlates with positive urine cultures in patients with suspected UTI and therefore could be used as an early non-invasive and easy-to-determine diagnostic biomarker for UTI. Consequently, using cathelicidin level as a diagnostic UTI biomarker could save time in waiting for urine culture results for confirming UTI diagnosis and limit unnecessary administration of antibiotics in the meantime. However, since local cathelicidin production during an acute UTI depends on a sufficient vitamin D serum concentration, the diagnostic potential of cathelicidin is limited to UTI patients with sufficient vitamin D levels, while those with vitamin D insufficiency might have false negative results (low urinary cathelicidin levels despite positive urine cultures). Furthermore, patients with urinary tract functional abnormalities such as VUR or obstruction might have elevated cathelicidin levels even without an acute infection. On the other hand, low urinary cathelicidin levels could predict recurrent UTIs despite the patient’s vitamin D status or underlying urinary tract functional abnormalities, since both patients with and those without vitamin D insufficiency and/or functional abnormalities might have insufficient cathelicidin production and therefore increased susceptibility for UTIs. Utilizing cathelicidin as a predictive biomarker of a recurrent UTI could limit unnecessary exposure to antibiotic prophylaxis and invasive diagnostic procedures in patients with a low risk of developing UTI recurrence. However, studies in this field are lacking and forthcoming studies should elucidate whether urinary cathelicidin level could predict UTI recurrence.

Furthermore, due to the emerging resistance of uropathogens to widely used antibiotics, discovery of new treatment strategies is urgent. Therapeutic agents targeting cathelicidin expression could be the answer, since cathelicidin is known for its bactericidal effect as well as innate and adaptive immunity enhancement. By inducing and activating local cathelicidin production, a local rather than a systemic effect could be achieved, offering a site-specific treatment. All of the investigated therapeutic agents in the reviewed studies, including vitamin D, estrogen, ceragenins, metformin and synthetic peptides such as CD4-PP, up-regulated cathelicidin production and consequently improved bactericidal activity and reduced bacterial load. However, studies investigating potential therapeutic agents that influence cathelicidin production are highly varied in their design, the therapeutic agents used and the parameters measured. As a result, although these studies show promise, they still require validation in larger prospective studies. In a conclusion, cathelicidin is a promising biomarker for both UTI diagnosis and prognosis, as well as a potential target of future UTI therapies. We hope that this review will inspire and encourage further research of this evolutionary conserved protein with multiple clinical potentials for improving the current management of urinary tract diseases.

## Figures and Tables

**Table 1:** Diagnostic and prognostic role of cathelicidin in urinary tract diseases.

Study [Reference Number]	Number of Subjects (Characteristics): -Subgroups	Method Used for Measuring Urinary or Serum Cathelicidin	Study Conclusions
Chromek et al. [7]	57 children: - 29 with acute UTI - 28 healthy controls	ELISA, urinary cathelicidin concentration	Cathelicidin’s production in the uroepithelium is increased during an acute UTI (*p* < 0.001) and therefore might be used as a diagnostic biomarker for UTI.
Nielsen et al. [21]	97 premenopausal adult women (27–46 years old): - 47 with an acute UTI - 50 healthy controls who had never had a UTI	ELISA, urinary cathelicidin concentration	Urinary cathelicidin level increases during an acute UTI (*p* = 0.0002) and therefore might be used as a diagnostic biomarker.
Van der Starre et al. [22]	137 adults (46–82 years old): - 45 with UTI and bacteremia - 46 with UTI, but without bacteremia - 46 controls without an acute infection	ELISA, urinary cathelicidin concentration	Urinary cathelicidin level is significantly elevated in an acute UTI and therefore might be used as a diagnostic biomarker (*p* < 0.0001). However, cathelicidin urinary level does not correlate with bacteremia occurance (*p* = 0.23).
Ovunc Hacihamdioglu et al. [23]	74 children (0.25–13 years): - 36 with an acute UTI - 38 healthy controls	ELISA, urinary cathelicidin concentration	Urinary cathelicidin might not be a reliable diagnostic biomarker for UTI in children with vitamin D insufficiency, since children with vitamin D insufficiency are not able to increase their urinary cathelicidin production during UTI (*p* > 0.05) opposed to those with sufficient vitamin D plasma levels (*p* = 0.001).
Caterino et al. [24]	40 patients with suspected UTI (36–78 years): - 13 with positive urine cultures - 27 with negative urine cultures	ELISA, urinary cathelicidin concentration	Since urinary cathelicidin level is not correlated with urine culture positivity (AUC = 0.50), it might not be applicable for UTI diagnosis for patients with vitamin D insufficiency.
Babikir et al. [25]	174 patients (children and adults): - 87 with an acute UTI - 87 healthy controls	ELISA, serum and urinary cathelicidin concentration	Both plasma and urinary cathelicidin levels might be used as early diagnostic biomarkers of UTI (*p* = 0.002 and *p* < 0.001, respectively).
Awadallah et al. [26]	90 adults (23.5–39.5 years old): - 50 with suspected and urine culture-positive UTI - 20 with suspected UTI, but with negative urine culture - 20 healthy controls	ELISA, urinary cathelicidin concentration	Urinary cathelicidin level correlates with urine culture positivity in suspected UTI patients (*p* < 0.001); therefore, it might be used as an early diagnostic biomarker for UTI.
Alhamedy et al. [27]	90 women (10–55 years old): - 60 with confirmed UTI - 30 healthy controls	ELISA, serum and urinary cathelicidin concentration	Both urinary and serum cathelicidin levels are elevated in patients with UTI compared with healthy controls (*p* < 0.001 and *p* < 0.0001, respectively), and therefore might assist UTI diagnosis.
Ali et al. [28]	60 adults (30–60 years old): - 30 with UTI - 30 healthy controls	ELISA, serum and urinary cathelicidin concentration	Both urinary and serum cathelicidin levels are elevated in patients with UTI compared with healthy controls (*p* < 0.05 for both urine and serum cathelicidin) and might be used as diagnostic biomarkers.
Krivošikova at al. [29]	148 children: - 98 children with UTI (aged 0.3–1.3 years) - 50 healthy controls (aged 0.5–5.2 years)	ELISA, urinary cathelicidin concentration	Children with a first febrile UTI have higher urinary cthelicidin levels than healthy children (*p* < 0.01), suggesting its potential as a UTI diagnostic biomarker.
Georgieva et al. [30]	120 children (aged 4.5–33.5 months): - 76 one month after UTI - 44 with congenital hydronephrosis but without a history of UTI	ELISA, serum cathelicidin concentration	Cathelicidin plasma level was lower in children one month after an acute UTI than in children without UTI history (*p* < 0.0001). Cathelicidin plasma level one month after the UTI did not prove to be a predictor of UTI recurrence (*p* > 0.05).
Lezhenko and Zakhar-chenko [31]	97 children (6–14 years old): - 77 with acute UTI, of whom 34 developed chronic UTI - 20 healthy controls	ELISA, serum cathelicidin concentration	Serum cathelicidin level is not a risk factor for developing chronic UTIs; however, serum vitamin D level seems to be associated with UTI recurrence.
Gupta et al. [32]	45 children (0.3–18.4 years old): - 30 patients with ureteropelvic junction obstruction - 15 healthy controls	ELISA, urinary cathelicidin concentration	Urinary cathelicidin is a potential diagnostic biomarker of obstructive uropathy (*p* < 0.001).
Gupta et al. [33]	13 patients with ureteropelvic junction obstruction (0.4–18.4 years)	ELISA, urinary cathelicidin concentration	Cathelicidin might not have a role in predicting clinical course after surgical repair of a urinary tract obstruction (*p* = 0.5417).
Gupta et al. [34]	43 children (0.4–18.0 years old): - 36 children with neurogenic bladder due to spinal dysraphism (6 with acute UTI, 18 with asymptomatic bacteriuria, 12 with sterile urine) - 17 healthy controls	ELISA, urinary cathelicidin concentration	Cathelicidin is elevated in uninfected children with neurogenic bladder compared to healthy children (*p* = 0.0006), probably due to uroepithelial abnormalities. Cathelicidin might not have a utility in differentiating acute UTI and asymptomatic bacteriuria in children with neurogenic bladder (*p* = 0.5617).
Colceriu et al. [36]	78 children (aged 1 month to 17 years): - 39 with VUR without an acute infection (9 with renal scarring) - 39 healthy controls	ELISA, urinary cathelicidin concentration	Cathelicidin might be a diagnostic marker of severe VUR (*p* = 0.02), as well as a predictive marker of renal scarring development in patients with VUR (*p* = 0.01).

**Table 2:** Therapeutic potential of agents targeting cathelicidin for the UTI treatment.

Study [Reference Number]	Therapeutic Agent Used in the Study	Study Findings
Herrting et al. [41]	Vitamin D	Oral vitamin D supplementation increases cathelicidin production during an acute UTI and consequently increases uroepithelial bactericidal activity against *E. coli*.
Luthje et al. [42]	Estradiol	Estrogen supplementation induces uroepithelial cathelicidin production, thereby enhancing its antimicrobial capacity and therefore could be used for preventing UTI in postmenopausal women.
Lindblad et al. [43]	NLRP-3 activator (UPEC strain CFT073)	Activation of NLRP3 by bacteria (UPEC strain CFT073) regulates the expression of cathelicidin in bladder epithelial cells and therefore is a potential target of preventive or curative UTI treatments.
Wang et al. [44]	BMAP-18	BMAP-18 incorporated with polyutrethane inhibits bacterial biofilm formation of common pathogens for catheter-related UTI, which makes it a promising therapeutic agent for preventing catheter-associated UTIs.
Wnorowska et al. [45]	Ceragenins CSA-13 and CSA-131	Ceragenins mimic endogenous AMPs and increase cathelicidin’s bactericidal activity, even against multi-drug-resistant *E. coli* strains.
White JK et al. [46]	Cathelicidin derived synthetic peptide CD4-PP	CD4-PP reduces uropathogen survival in vitro by a direct bactericidal effect, preventing biofilm formation and inducing cathelicidin production.
Mohanty et al. [47]	2-oxoglutarate analogue DMOG (HIF-hydroxylase inhibitor)	DMOG enhances HIF-1, which induces cathelicidin and consequently reduces the bacterial load in in vitro and in vivo diabetic UTI models.
Majhi et al. [48]	Metformin	Metformin stimulates cathelicidin production, therefore strengthening the innate immunity of uroepithelium and resulting in reduced bacterial load.
Luthje et al. [49]	Simvastatin	Simvastatin induces cathelicidin expression in bladder epithelial cells and therefore might have a role in preventing or treating UTI.
Schwarz et al. [50]	Insulin	Activation of insulin receptor in uroepithelial cells induces cathelicidin production and thus offers new possibilities for preventive or curative UTI treatment.

## Data Availability

No new data were created in this study.
